# Evidence that 17alpha-estradiol is biologically active in the uterine tissue: Antiuterotonic and antiuterotrophic action

**DOI:** 10.1186/1477-7827-3-30

**Published:** 2005-07-21

**Authors:** Mercedes Perusquía, Erika Navarrete

**Affiliations:** 1 Department of Cell Biology and Physiology, Institute for Biomedical Research, National Autonomous University of Mexico (UNAM), Apartado Postal 70228, Mexico City 04510, Mexico

## Abstract

**Background:**

17alpha-Estradiol has been considered as the hormonally inactive isomer of 17beta-estradiol. Recently, nongenomic (smooth muscle relaxation) and genomic (light estrogenic activity) effects of 17alpha-estradiol have been reported, but no reports have yet determined its possible antiestrogenic activity. Therefore, this study investigated: the nongenomic action of 17alpha-estradiol on uterine contractile activity and its potential agonist-antagonist activity on uterine growth.

**Methods:**

Uterine rings from rats were isometrically recorded. Different concentrations (0.2–200 microM) of 17alpha-estradiol were tested on spontaneous contraction and equimolarly compared with 17beta-estradiol. To examine the mechanism of 17alpha-estradiol action, its effect was studied in presence of beta2-antagonist (propranolol), antiestrogens (tamoxifen and ICI 182,780) or inhibitors of protein synthesis (cycloheximide) and transcription (actinomycin D). Moreover, contractions induced by high potassium (KCl) solution or calcium in depolarized tissues by KCl-calcium free solution were exposed to 17alpha-estradiol. Collaterally, we performed an uterotrophic assay in adult ovariectomized rats measuring the uterine wet weight. The administration for three days of 0.3 microM/day/Kg 17beta-estradiol was equimolarly compared with the response produced by 17alpha-estradiol. Antiuterotrophic activity was assayed by administration of 0.3 microM/day/Kg 17beta-estradiol and various doses ratios (1:1, 1:3, 1:5, and 1:100) of 17alpha-estradiol.

**Results:**

The estradiol isomers elicited an immediate relaxation, concentration-dependent and reversible on spontaneous contraction. 17alpha-Estradiol presented lower potency than 17beta-estradiol although it did not antagonize 17beta-estradiol-induced relaxation. Relaxation to 17alpha-estradiol was not inhibited by propranolol, tamoxifen, ICI 182,780, cycloheximide or actinomycin D. The KCl contractions were also sensitive to 17alpha-estradiol-induced relaxation and calcium contractions in depolarized tissues were markedly prevented by 17alpha-estradiol, implying a reduction of extracellular calcium influx through voltage-operated calcium channels (VOCCs). Uterotrophic assay detected significant increase in uterine weight using 17alpha-estradiol, which was significantly minor as compared with 17beta-estradiol. 17alpha-Estradiol, at all doses ratios, significantly antagonized the hypertrophic response of 17beta-estradiol.

**Conclusion:**

17alpha-Estradiol induces a relaxing effect, which may be independent of the classical estrogen receptor, nongenomic action, apparently mediated by inactivation of VOCCs. 17alpha-Estradiol is also a weak estrogen agonist (uterotrophic response); likewise, 17alpha-estradiol may act as an antiestrogen (antiuterotrophic response). The overall data document a nongenomic relaxing action and a novel antiestrogenic action of 17alpha-estradiol, which are relevant in estrogen-mediated uterine physiology.

## Background

17α-Estradiol (17α-E2) has long been considered as the hormonally inactive isomer of 17β-estradiol (17β-E2) useful in determining the hormonal specificity of response to 17β-E2 [1,2]. Consequently, it has been generally accepted that 17α-E2 is devoid of genomic estrogenic effects [3-6]. Nevertheless, in the past few years it has been documented that 17α-E2 may induce genomic effects such as partial estrogenic activity [7-11]. In addition, this estrogen possesses important nongenomic (membrane) actions by inducing neuroprotective [12,13] and mitochondrial protective [14] effects, as well as relaxing effects in isolated vascular [15-17], uterine [18] and urinary [19] smooth muscle. In this respect it is reasonable to assume that, 17α-E2 may play a relevant physiological role, but little attention has been paid to examine its potential regulatory function.

On the other hand, the available data have shown that 17α-E2 is the predominant estrogen in some mammals, whereas only few studies exist concerning the detection of 17α-E2 in humans which has only been found in the urine and serum at low concentrations [reviewed in [20,21]]. However, is important to highlight that 17α-E2 is used as an ingredient of estrogen replacement therapy and hormone replacement therapy applied in the treatment of peri- and post-menopausal women [22].

Therefore, the present study was designed to explore the feasible actions of 17α-E2 in the uterine tissue. Specifically, we have examined the possible effects of this hormone on both nongenomic and genomic actions in the rat uterus: (1) some studies were performed on uterine contractile activity by using a well established isometric system for isolated tissue. The effects were observed by application of 17α-E2 on the spontaneous and KCl-induced myometrial contraction. The mechanism of action of 17α-E2 was delineated to determine if its potential relaxing effect on uterine contractility is genomically mediated or if this estrogen is interacting with membrane proteins (calcium channels and/or adrenoceptors); and (2) on the basis that some natural stereoisomers, as in the case of testosterone and epitestosterone which elicit nongenomic uterine relaxing action [23] and only epitestosterone has antiandrogenic activity [24-26], the estradiol isomers, 17α- and 17β-E2, should also induce agonist-antagonist activities. Thus, we have quantified estrogenicity and antiestrogenicity in a classical sense, determining these actions on uterine wet weigh. Accordingly, this study set out to investigate the potential antagonist (antiestrogenic) activity of 17α-E2 on the uterotrophic response induced by 17β-E2.

## Methods

### Animals

Female Wistar rats weighing 180–220 g were obtained from Charles River Breeding Laboratories (Wilmington, MA), housed in our animal facility under controlled lighting (lights-on from 0700–1900 h) and temperature (21°C) conditions, and given *ad libitum *water and food. The project was approved by our Animal Care Committee, and experiments were conducted in accordance with the published Guiding Principles in the Care and Use of Animals approved by the American Physiological Society. The vaginal smears of these animals were inspected daily for 2 weeks, and animals showing regular 4-day estrous cycle were selected on the day of diestrus.

### Myometrium contractile activity

The rats were killed and the uterine tissues were immediately removed and transferred to warmed (37°C), oxygenated (O_2_/CO_2 _95:5) Krebs-bicarbonate solution of the following composition (mM): NaHCO_3 _(25), NaCl (119), KCl (4.6), KH_2_PO_4 _(1.2), MgSO_4 _(1.2), CaCl_2 _(1.5) and glucose (12), with the pH adjusted to 7.4. The uterine horns were isolated, cleaned of surrounding fat and loose connective tissue, and transversally bisected into two rings, approximately 1 cm in length.

The uterine rings were placed vertically in a 10 ml tissue chamber and bathed in Krebs-bicarbonate solution, under optimum resting force of 10 mN (1 g tension), and allowed for 30 min before starting the experiment. The contractile response of each tissue was recorded isometrically using transducers (FTO3C; Grass Instruments, Quincy, MA) connected to a polygraph (79; Grass Instruments).

After a stabilization period (1 h) of the tissues in Krebs-bicarbonate solution, the spontaneous uterine contraction was recorded for 10 min, and this was taken as the control value (100%). Immediately, 17α-E2, dissolved in absolute ethanol and added to the bath tissue in a final volume of 0.1%, was tested by adding increasing concentrations in a non-accumulative manner (in concentrations ranging from 0.2 to 200 μM; each concentration never exceeded 0.1% v/v of vehicle). Only one concentration was used for each uterine ring from different animals. The estrogen effects were also recorded for 10 min, and the response was compared with the control. In a separate group of experiments, uterine tissue was exposed to vehicle alone (0.1 % ethanol). The concentration-response curves to estrogen on spontaneous contraction were plotted, and the medium inhibitory concentration (IC_50_; value for estrogen concentration at which 50% of the maximum inhibition of uterine contraction was achieved) was calculated as described by Litchfield and Wilcoxon [27]. In order to evaluate the inhibitory response of 17α-E2, its potency was compared with that of 17β-E2, which was used as a positive control under the same experimental conditions.

In a series of experiments the tissues were incubated with: antiestrogens, 1 μM tamoxifen (estrogen receptor antagonist) or 1 μM ICI 182,780 (the pure estrogen receptor antagonist), as well as 10 μM actinomycin D (inhibitor of gene transcription) or 100 μM cycloheximide (inhibitor of protein synthesis), 30 min before of 17α-E2 addition at 89.39 μM (IC_50_). The effect of 17α-E2 was evaluated for 10 min in presence of each drug, and compared with the effect that this estrogen produces alone. In addition, the final volume of vehicle (0.1% absolute ethanol) by each substance added was observed in independent experiments.

To examine the potential blocking effect of 17α-E2 on 17β-E2-induced inhibition, the effect of both 17β- and 17α-E2 was also studied on the spontaneous contraction. The tissue was pretreated with 17β-E2 at 8.42 μM (IC_50_), 10 min before of 17α-E2 addition (89.39 μM; IC_50_), and then the response was recorded for 10 min. The relaxing response induced by both estrogens was evaluated and compared when 17α-E2 was not present. In the same way, the opposite treatment (89.39 μM 17α-E2 before 8.42 μM 17β-E2 addition) was also determined.

The effect of 17β- and 17α-E2 was also studied on the contraction induced by high potassium (KCl 40 mM) solution, after replacing normal Krebs-bicarbonate solution with an equimolar substitution of 40 mM KCl and 84 mM NaCl. Thus, KCl solution induces a tonic contraction and after a stable contractile tension was attained (~20 min) each estradiol, 17β- or 17α-E2, at 200 μM (highest concentration tested on spontaneous contraction) was separately added and the effect was recorded for 10 min. This effect was compared with their inhibitory responses at 200 μM on spontaneous contraction. Finally, the tissues were washed and a next contraction induced by KCl was observed for 60 min to check the tissue recovery. Collaterally, the contraction induced by KCl was exposed to vehicle (0.1% ethanol) alone.

Some additional experiments were carried out to analyze the possible interaction of 17α-E2 with the β-adrenoceptors. For this purpose, 5 μM of noradrenaline (β_2_-adrenoceptor agonist) was applied 10 min after the KCl stimulus and its relaxing effect was evaluated for 10 min. Previously, we observed that the relaxation induced by 5 μM of noradrenaline on KCl contraction was completely blocked when the tissues were preincubated 5 min before with 20 μM propranolol (β_2_-adrenoceptor antagonist). Following the same protocol, the effect of 17α-E2 at 89.39 μM was observed with 20 μM propranolol preincubation.

Other uterine rings were depolarized with a high potassium-calcium free solution; depolarizing solution (KCl) modified by addition of 2 mM EGTA and without CaCl_2_. A transient contraction was obtained by high potassium-calcium free solution and when the baseline was reached, 1 mM CaCl_2 _was added to evoke a tonic contraction, which was recorded for 20 min. This process was repeated until a reproducible response was obtained (control); then, the tissues were preincubated with 17α-E2 at 89.39 μM (IC_50 _on spontaneous contraction) 5 min before the addition of CaCl_2 _at 1 mM. Under this conditions, the contraction induced by CaCl_2 _was recorded for 20 min in the presence of 17α-E2, which was compared with the control. Subsequently, the tissues were washed out and a CaCl_2_-induced contraction was elicited again. The washout was done after all calcium contractions, three times, with depolarizing free-calcium solution. This protocol was used to test the potential voltage-operated calcium channel blocking properties of 17α-E2.

### Uterotrophic and antiuterotrophic activity

Other rats in diestrus were ovariectomized under ether anesthesia. Fifteen days later, the animals were divided into seven groups (n ≥ 6 each), and they were injected subcutaneously, once daily for three days, with 0.4 ml/Kg body weight of vehicle (corn oil; group I), 0.3 μmol/Kg body weight of 17β-E_2 _or 17α-E2 (group II and III, respectively), and in combination with varying concomitant doses of 17β-E2/17α-E2 (μmol/day/Kg body weight): 1:1 (0.3:0.3; group IV), 1:3 (0.3:0.9; group V), 1:5 (0.3:1.5; group VI) and 1:100 (0.3:30; group VII), all dissolved and administered in 0.4 ml/day/Kg of the vehicle. The rats were weighed 24 h after the last dose, and vaginal smears were taken and examined under the microscope. Autopsy was performed and the uteri were carefully dissected out, blotted and the organ wet weights were recorded.

### Data presentation and statistical analysis

The total contractile activity (the area under the curve inscribed by the frequency and amplitude of contraction) was measured during each 10-min interval by using PolyView system 2.1 (Grass Instruments Division/Astro-Med. Inc, West Warwick, RI) data acquisition and playback software. The data for compound action on the uterine contractility were calculated as mean value of more than 6 independent determinations, each from different experiments and expressed as percentages ± SEM. In order to evaluate the inhibitory response of 17α-E2, its potency was compared with the inhibitory effect of induced by 17β-E2. The gain in uterine weight of each group was calculated as mg uterine weight/100 g body weight. 17α-E2 (group III) was compared with the vehicle (group I) and 17β-E2 (group II) effect. The treated groups at different doses range of 17β-/17α-E2 were compared with the uterotrophic effect of 17β-E2 alone (uterine growth induced at 0.3 μmol/day/Kg body weight = 100%). Non-paired Student's t-test was utilized to compare the responses between two groups. We used two-way-ANOVA to compare the concentration-response curves in isolated tissues. For multiple comparisons, one-way-ANOVA with Bonferroni correction was used for antiuterotrophic assay. A value of P < 0.05 was accepted as statistical significance.

### Chemicals

The following compounds were used: 1,3,5(10)-estratriene-3,17β-diol (17β-estradiol; 17β-E2), 1,3,5(10)-estratriene-3,17α-diol (17α-estradiol; 17α-E2), tamoxifen (estrogen receptor antagonist), ICI 182,780 (the pure estrogen receptor antagonist), actinomycin D (transcription inhibitor), cycloheximide (protein synthesis inhibitor), propranolol hydrochloride (β_2_-adrenoceptor antagonist; P) and noradrenaline hydrochloride (noradrenaline; NA). With the exception of ICI 182,780 (obtained from Tocris Cookson, Ellisville, MO, USA), the remaining compounds used in the present study were all purchased from Sigma Chemical Co., St. Louis MO, USA. In isolated tissue preparations, all compounds were prepared as stock solution (for each concentration) in absolute ethanol and added to the bath chamber in a final volume of 0.1% (absolute ethanol), except for NA and P which were dissolved in distilled water. Actinomycin D and NA were kept in the dark until use in order to avoid light-induced degradation. With respect to the in vivo experiments, the estrogens were dissolved and administered in the same volume of corn oil (0.4 ml/Kg).

## Results

### Inhibitory effect of 17α-E2 on spontaneous contractility

**Figure 1:**
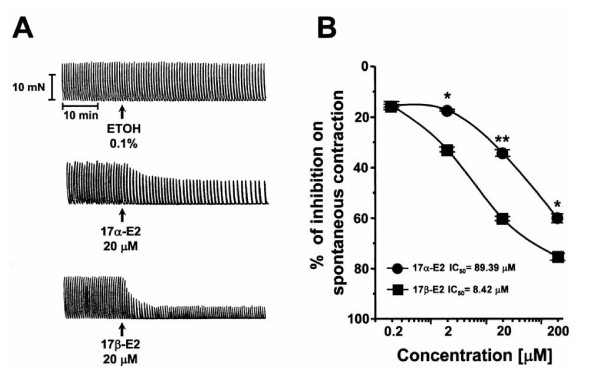
**Inhibitory effect of 17α- and 17β-E2 on spontaneous uterine contractile activity of rats in diestrus**. A) The vehicle utilized to dissolve each estradiol, ethanol (ETOH 0.1%), did not significantly modify (P > 0.05) the spontaneous contractility. 17α- and 17β-E2 induce inhibition of contractile activity. Note their different efficacy when they are added at the same concentration (20 μM). B) Concentration-response curves to 17α- and 17β-E2 on spontaneous contractility, which were significantly different (P < 0.0005) between them. Each point represents the mean ± SEM of six independent experiments. Statistical significance between concentrations: *P < 0.005, **P < 0.0005. The effect induced by both estradiol isomers at all concentrations tested were significantly different (P < 0.0005) from the vehicle control.

As shown in Fig. 1A, the vehicle of estrogens, ethanol (0.1%; a final volume identical to those added as solvent for estrogens), did not significantly modify spontaneous uterine contractility (2.95 ± 0.25% of inhibition, n = 6, P > 0.05). 17α- and 17β-E2 caused a concentration-dependent inhibition of spontaneous uterine contractility (Fig. 1B), with an IC_50 _value of 89.39 and 8.42 μM, respectively. Therefore, the effect of 17β-E2 was 10.6 fold more potent than 17α-E2 to inhibit the spontaneous uterine contractility. As shown in Fig. 1B, the concentration-response curves to 17α- and 17β-E2 were significantly different between them (P < 0.0005). The inhibitory effect of 17α-E2 and its 17β isomer was observed within 1 min after the uterine tissue was exposed to the estrogen (Fig. 1A) and the spontaneous contractility was reversed after estrogen was removed (washed out) from the tissue. We also observed that addition of 89. 39 μM 17α-E2 did not stop the previous inhibitory effect induced by 17β-E2 at 8.42 μM. On the contrary, 17β-E2-induced inhibition (30.6 ± 1.08%) was significantly enhanced after addition of 17α-E2 (70.53 ± 1.77% of inhibition; P < 0.0005). Furthermore, the opposite treatment (17α-E2 before 17β-E2 addition) revealed that the inhibitory effect of 17β-E2 was not antagonized by 17α-E2 pretreatment, but was also significantly enhanced (73.97 ± 1.97% of inhibition; n = 6, P < 0.0005). Thus, this observation implies that both inhibitory effects were synergized.

### Effect of estrogens on KCl-induced uterine contraction

**Figure 2:**
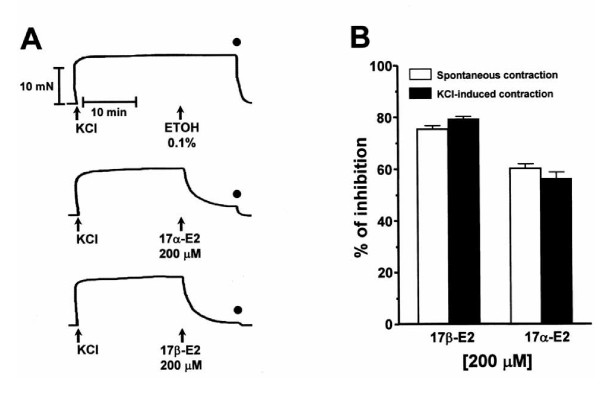
**Effect induced by 17α- and 17β-E2 on uterine contraction achieved by high potassium (KCl 40 mM)**. A) The vehicle of estrogens, ethanol (ETOH 0.1%), did not significantly modify KCl-induced contraction (P > 0.05) whereas this tonic contraction was significantly inhibited by 17α- or 17β-E2 and this response was also significantly different from the vehicle control (P < 0.0005). The black circles represent the time of washout. B) Comparison of inhibitory effect at equimolar concentration (200 μM) of 17α- and 17β-E2 on spontaneous and KCl(40 mM)-induced contraction. The student's t test demonstrated that the relaxing efficacy of 17α- and 17β-E2 was not significantly different (P > 0.05) on spontaneous or KCl-induced contraction. The plotted values represent the mean ± SEM of six independent experiments.

The tonic contraction induced by KCl was also inhibited by each estradiol at the highest concentration tested on spontaneous contraction (200 μM), the development of the relaxing effect in precontracted tissues also started within a few seconds (~30 sec) after addition of each estrogen (Fig. 2A). Likewise, after washout the amplitude and tone of the next KCl-induced contraction was totally recovered. As shown in Fig. 2B, the relaxing efficacy of 17α- and 17β-E2 was not significantly different in both spontaneous and KCl-induced contraction; however, the relaxing effect induced by 17β-E2 was higher than that induced by 17α-E2 in both contractile responses. The vehicle of estrogens (ethanol 0.1%) did not significantly affect (1.39 ± 0.17% of relaxation; n = 6, P > 0.05) the tone of KCl contraction, but the effect induced by each estradiol was significantly different (P < 0.0005) from the vehicle control (Fig 2A).

### Inhibitory effect of 17α-E2 in presence of different drugs

**Figure 3:**
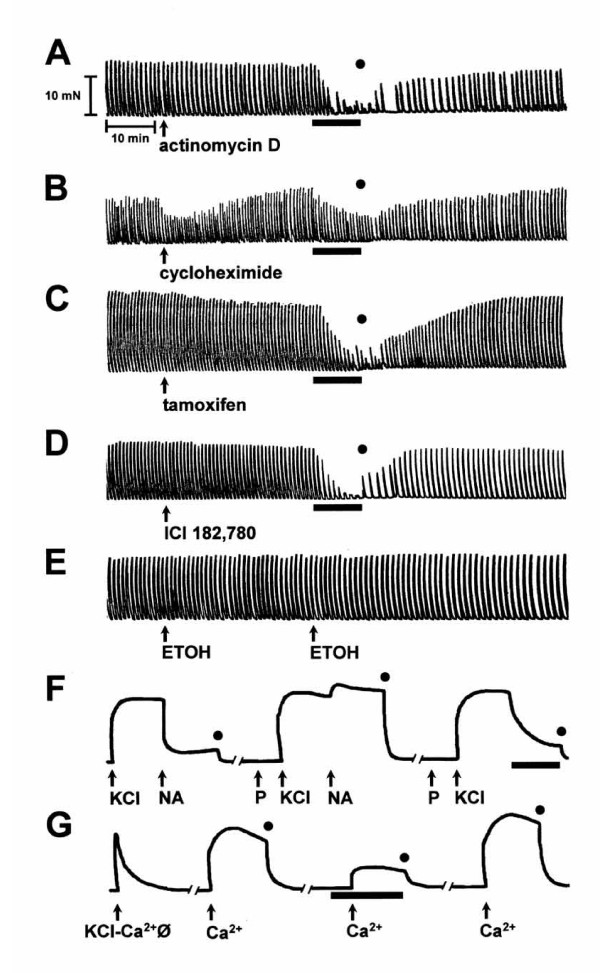
**Original recordings demonstrating typical effect of 17α-E2 on uterine contractions. **The inhibitory effect of 17α-E2 on spontaneous contractility was not blocked by preincubation with inhibitors of transcription, 10 μM actinomycin D (A) and protein synthesis, 100 μM cycloheximide (B) or antiestrogens, 1 μM tamoxifen or 1 μM ICI 182,780 (C and D, respectively). The vehicle utilized to add each compound, absolute ethanol (ETOH 0.1%), did not affect (P > 0.05) the spontaneous contractility (E). KCl-induced contraction was inhibited by addition of 5 μM noradrenaline (NA), which was antagonized by preincubation with β_2_-adrenoceptor antagonist, 20 μM propranolol (P), while 17α-E2-induced relaxation was not antagonized by P (F). The relaxing efficacy of estrogen was not modified in tissues pretreated with these blocking agents. The calcium-induced contraction at 1 mM (Ca^2+^) in tissues previously depolarized by high potassium-calcium free solution (KCl-Ca^2+^φ) was notably prevented by 17α-E2 (G). The solid black line indicates the incubation time with 17α-E2 at 89.39 μM. Note the contraction recovery after washout (represented by the black circles), showing that the estrogen effect was reversible.

These results are illustrated in Fig. 3. The inhibitory effect elicited by 17α-E2 (89.39 μM) was not blocked by gene transcription (Fig. 3A) or protein synthesis (Fig. 3B) inhibitor. Likewise, we observed that the estrogen receptor antagonists (tamoxifen or ICI 182,780) failed to affect 17α-E2-induced uterine inhibition (Fig. 3C and 3D, respectively). Moreover, the response to 17α-E2 has rapid time-courses, and the spontaneous uterine contractility was totally recovered after estrogen was removed from the tissue (washout). As shown in Fig. 3E, the control vehicle (absolute ethanol; final volume 0.1%) by each drug (inhibitor and estrogen) did not significantly modify the spontaneous contractility (3.47 ± 1.14% of inhibition; P > 0.05). Propranolol at 20 μM blocked the inhibitory effect of 5 μM noradrenaline, but this β_2_-adrenoceptor antagonist (at the same concentration) did not block the relaxation induced by 17α-E2 on KCl-induced contraction (Fig. 3F).

### Effect of 17α-E2 on calcium-induced contraction

The uterine tissues were depolarized by high potassium-calcium free solution. Under these experimental conditions, a tonic contraction was induced by CaCl_2 _(1 mM), which was antagonized when tissues were preincubated with 89.39 μM 17α-E2 (71.03 ± 1.93% of inhibition, n = 6), observing that the amplitude was significantly decreased (Fig. 3G). The calcium antagonistic effect induced by 17α-E2 was reversible upon washing out the tissue and removing the estrogen (see Fig. 3G; third CaCl_2 _addition). Importantly, the prevention of this calcium contraction by 17α-E2 turned out significantly higher (P < 0.0005) than its inhibitory effect on spontaneous contraction (52.12 ± 2.05 %). The final volume of estrogen vehicle (0.1% absolute ethanol) did not significantly prevent calcium-induced contraction (3.55 ± 0.45%; n = 6, P > 0.05).

### Agonistic and antagonistic activity on uterine growth

**Figure 4:**
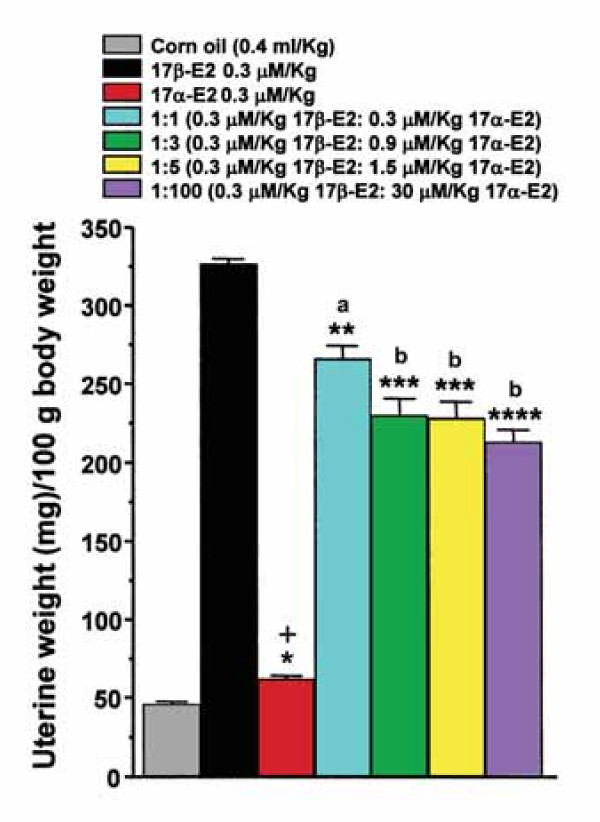
**Action of 17α- and 17β-E2 on uterine mass in adult ovariectomized rats**. 17α-E2 significantly increased the uterine weight as compared with the vehicle (corn oil; *P < 0.05), although this increment was significantly less than those of 17β-E2 (^+^P < 0.00005). In rats treated with 17β-E2 plus different doses of 17α-E2, less uterotrophic effect of 17β-E2 was evident. Statistical significance: **P < 0.005, ***P < 0.0005, ****P < 0.0001. The analysis shows that the treatment 1:1 (a) was different from 1:3, 1:5 and 1:100 (b; P < 0.05), which were not different between them (b; P > 0.05). Each bar represents the mean ± SEM, n = 6 rats per group.

17α-E2 action on the gain uterine weight was significantly different (P < 0.05) to vehicle group (corn oil), thus this hormone presented a light uterotrophic activity in contrast with the potent uterotrophic activity induced by its 17β isomer at the same dose (Fig. 4). However, the antiuterotrophic activity of 17α-E2 was assayed and we observed that this hormone had a significant antagonistic effect on the uterotrophic action of 17β-E2 at all doses ratios (Table 1 and Fig. 4). Additionally, the vaginal smears from rats treated with 17β-E2 showed that the vaginal cornification was decreased as 17α-E2 dose was increased (data not shown).

**Table 1:** Antiuterotrophic activity of 17α-E2 in adult ovariectomized rats.

**Dosage of 17α-E2 (μmol/day/Kg body weight)**	**Ratio^**a **^of 17β-E2:17α-E2**	**Uterine wet weight/100 g**^**b**^	**% inhibition**^**c**^
0.3	1:1	266.1 ± 8.2	18.55
0.9	1:3	229.9 ± 10.7	29.63
1.5	1:5	228.3 ± 10.4	30.11
30.0	1:100	213.1 ± 7.8	34.77

^a^Fixed dose of 17β-E2 used = 0.3 μmol/day/Kg body weight.

^b^Data from six independent experiments ± SEM (six rats per group).

^c^As compared with uterotrophic effect of 17β-E2.

## Discussion

The results of the present study demonstrate that 17α-E2 is a natural estrogen hormonally active in the rat uterine tissue. Our findings show that this estrogen is capable of inducing both nongenomic (antiuterotonic effect) and genomic (estrogenic/antiestrogenic effect) action. This evidence may, in fact, account for its potential regulatory biological function.

In particular, 17α-E2 elicits inhibition of uterine contractile activity by inducing antiuterotonic responses on spontaneous and KCl-induced contraction. These findings are in line with a previous study in isolated rat uterus, which reported that 17α-E2 has the ability to produce relaxation on contractions induced by KCl, calcium and vanadate [18]. We observed that 17α-E2-induced inhibition was significantly different as compared to its 17β isomer, with about 10-fold lower potency than 17β-E2, implying a partial agonist effect of 17α-E2 on uterine contractile activity. Nevertheless, this inhibitory effect could be relevant to promote uterine quiescence during pregnancy. Admittedly, the concentrations of the inhibitory responses to 17α- and 17β-E2 may be in pharmacological ranges; however, these are close to the therapeutic doses used.

Regarding the mode of action of 17α-E2-induced uterine relaxation, it is important to emphasize that the instantaneous relaxing effect of 17α-E2, plus the evidence that the effect disappears after the estrogen is removed from the tissue, are uncharacteristic of classical genomic activities. Thus, this effect is presumably through nongenomic (membrane) actions. This idea is supported by the fact that the observed effect of 17α-E2 on uterine contractility occurred within 1 min of its addition, and 1 min is not enough time for genomic effects to occur [28]. Another approach to discriminate between genomic and nongenomic action is to use antihormones that bind to the intracellular steroid receptor, but that do not block the rapid membrane effects. Our results show that 17α-E2 on rat uterus does not seem to be mediated by genomic events since the antiestrogens, tamoxifen or ICI 182,780, and the transcriptional and translational inhibitors did not antagonize the uterine inhibitory effect of 17α-E2. This observation is in partial agreement with the study of Gutiérrez and coworkers [18], where it was also found that the relaxation induced by 17α-E2 on KCl-induced contraction was not blocked by tamoxifen, but differed by the fact that 17α-E2-induced relaxation was blocked by cycloheximide and actinomycin D, where an interaction with transcriptional ways was suggested. This apparent discrepancy may be explained because their evaluation was done under special experimental conditions; rats were estrogen-primed for 24 h. In this way, the administration of 17β-E2 results in changes of density and distribution of several receptors (proteins), called estrogen-dependent receptors, which are enhanced under conditions of estrogen dominance and, consequently, this treatment may modify the physiological response. In contrast, our data were obtained on spontaneous contractility of rats in diestrus, a model close to the physiological condition. Nevertheless, it should be kept in mind that the relaxing effect of 17α-E2 is too fast and reversible, a point of discussion to distinguish if the transcriptional process is or not involved.

Indeed, our findings indicate that 17α-E2 has a nongenomic action to induce myometrial relaxation, as previously reported to progestins and androgens in rat [29] and humans [30,31]. Likewise, our study has shown different sensitivity of uterine tissue to 17α- and 17β-E2-induced relaxation, suggesting a specific relaxing efficacy for each isomer. In this sense, we have reported before the presence of large differences in uterine relaxing potency in a series of closely related steroids, such as androgens and progestins, some of them without effect [29-32], pointing to a defined structure-activity relationship. Nevertheless, it is important to consider that not only is the uterine muscle the target of 17α-E2 to induce relaxation but also other types of smooth muscles such as those in the blood vessels [15-17] and bladder [19] are relaxed by this estrogen.

In view that the uterine relaxing effect of 17α-E2 can be explained by a nongenomic action, this effect may be associated with different sites of action at the cell surface (e.g. membrane proteins). In this respect, the possibility of an interaction of 17α-E2 with inhibitory β_2_-adrenoceptors can be dismissed since its specific antagonist (propranolol) did not block the uterine relaxing effect of 17α-E2. This evidence indicates that 17α-E2 induced a nonadrenergic inhibitory response on uterine contractility.

Since the involvement of β_2_-adrenoceptors seems improbable, the possibility has to be discussed finally that the nongenomic-relaxing effect of 17α-E2 involves a diminution on the intracellular concentration of calcium in the smooth muscle cells. In this connection, our preliminary results show that the efficacy induced by 17α-E2 was more prominent to antagonize contractions induced by calcium in depolarized tissues than to inhibit the spontaneous contractility. In this context, it is known that KCl depolarizes the membrane and opens voltage-operated calcium channels, resulting in calcium entry. Consequently, the inhibition induced by 17α-E2 in uterine rings precontracted by KCl and the marked antagonism of calcium entry in depolarized tissues are implying a direct reduction of extracellular calcium influx by producing inactivation of voltage-operated calcium channels. This hypothesis has also been proposed to the nongenomic relaxing effect of progestins and androgens in uterine [29-32] and vascular [33] smooth muscle. In support of this view, it has been reported that 17α-E2 might behave as a calcium channel antagonist in vascular cells [17,34] and this estrogen may also induce a reduction on voltage-dependent calcium currents in vascular smooth muscle cells [35,36]. Obviously, further patch-clamp experiments on uterine smooth muscle cells will be required to evaluate this issue.

It is also tempting to suggest that the nongenomic relaxing effect of 17α-E2 could be mediated through a subpopulation of the classical estrogen receptor (ER), ERα and ERβ, that is located at the plasma membrane [37,38] and specifically identified in caveolae and cell membranes from endothelial cells [39]. However, this possibility appears not to be supported by the fact that endogenous membrane and nuclear ER was found to be the same protein [40] and a much weaker affinity of 17α-E2 to the human ER compared to 17β-E2 has been shown [41]. In this context, our experimental evidence that two ER antagonists (tamoxifen or ICI 182,780) did not block 17α-E2-induced uterine relaxation implies that the estrogen binding site at the myometrial cell membrane could be a protein unrelated to the ERα or ERβ. Thus, the present findings indicate that 17α-E2 also acts at the cell surface of myometrial cells to initiate rapid, nongenomic, responses and this action may be mediated by interaction of estrogen with calcium channel proteins, which produces inactivation of voltage-operated calcium channels. Taken together these findings, it is important to consider that a nonspecific effect may also occur for the uterine relaxing effect of 17α-E2, such as changes in membrane fluidity.

Although 17α-E2 had been considered without estrogenic activity [3-6], we confirmed that 17α-E2 presents a significant weak estrogenic activity by inducing increase on uterine weight; this activity is much less potent than that induced by 17β-E2 and did not achieve full estrogenicity at higher doses. Consistent with this observation, it has also been reported that 17α-E2 induces light estrogenic activity [7-11]. However, to our knowledge, no evidence had been shown on the action of 17α-E2 as antagonist.

Remarkably, we have now demonstrated that 17α-E2 possesses antiestrogenic properties by antagonizing the uterotrophic response of 17β-E2, and we believe it plays an important role in estrogen-mediated uterine physiology. Furthermore, there is also the possibility that 17α-E2 should induce antiestrogenic activity in several tissues as well as in other species, including human; however, additional experiments are needed in order to address this question. The uterotrophic response of 17β-E2 was significantly antagonized by 17α-E2 at all doses ratios, although its antagonism was not dose-dependent at a dosage of 0.9 to 30 μM/Kg, with a maximal effect of ~30% of inhibition. Indeed, this may indicate that 17α-E2 acts as a partial antagonist of ER. Interestingly, the antiuterotrophic efficacy of 17α-E2 turned out similar to the antiuterotrophic action of tamoxifen, in a range of doses ratios from 1:2 to 1:10, as previously reported [42].

It is of note that the reduced efficacy of 17α-E2 to induce uterine relaxation and estrogenic activity, as compared with 17β-E2, is similar to that induced by tamoxifen, which deserves further consideration. In the first instance, tamoxifen is also capable of eliciting uterine relaxation [43-45]; consequently, this antiestrogen has been reported to have some nonreceptor-mediated effects [46] by unclear mechanisms. Moreover, since the ER is essential for 17β-E2-induced uterine proliferative responses [47], the present data suggest that 17α-E2 appears to regulate its slight agonist (estrogenic) and antagonist (antiestrogenic) activity through the same mechanism as tamoxifen. Recently, it has been documented that tamoxifen displays partial agonist-antagonist activities in different tissues and cells, and these differences may be related to the milieu of ER coactivators and corepressors in these tissues. The ER has two transcriptional activation domains, AF-1 and AF-2; thus, AF-1 activity is stimulated by tamoxifen binding to induce its partial agonist activity but AF-2 is inhibited when tamoxifen acts as antagonist [48-51]. With this line of evidence, could be reasonable to speculate that the mode of action of tamoxifen could be analogous for the agonist and antagonist properties of 17α-E2. Obviously, further studies that fall beyond the scope of the present investigation will be required to explore the agonistic-antagonistic mechanism of 17α-E2.

The present study revealed that both estradiol isomers, 17α- and 17β-E2, possess the same nongenomic action by inducing uterine relaxation but 17α-E2 is incapable of antagonizing the 17β-E2-induced uterine relaxation. Collaterally, these two isomers may also induce the same genomic action by eliciting an estrogenic uterotrophic response; however, the estrogenic activity of 17β-E2 is antagonized by 17α-E2. In this context, this evidence is correlated with the biological action emerged to other natural isomers, such as testosterone and epitestosterone which present the same nongenomic relaxing action [23], but only epitestosterone possesses a genomic antiandrogenic activity [24-26].

Finally, we also noted that the different structural conformation of both estradiol isomers could be important to induce diverse responses. The differences between both molecules is the α/*trans *or β/*cis *configuration at C-17. Thus, estrogen antagonistic activity of 17α-E2 could be presumed by its α/*trans *configuration, in contrast to the β/*cis *which is responsible for the marked antiuterotonic and estrogenic activity induced by 17β-E2.

## Conclusion

The preliminary investigation presented here reveals interesting features of the uterine function regulated by 17α-E2. The data indicate that this estrogen is an agonist inducing nongenomically mediated uterine relaxation, but also appears to have mixed agonist-antagonist activity on uterine growth, presumably through genomic processes. This study shows that a rapid nongenomic action (antiuterotonic response) of 17α-E2 takes place before its genomic action (uterotrophic-antiuterotrophic response). Additionally, this evidence could account for our knowledge of effects produced by 17α-E2 and some sulfate derivatives, which are components applied in the treatment of peri- and post-menopausal women.

## Authors' contributions

MP conceived and designed the study and wrote the article, and was also involved in acquisition, analysis and interpretation of data. EN carried out the in vitro studies, performed the statistical analysis and helped to draft the manuscript. The two authors read and approved the final manuscript.
